# Designing verbal autopsy studies

**DOI:** 10.1186/1478-7954-8-19

**Published:** 2010-06-23

**Authors:** Gary King, Ying Lu, Kenji Shibuya

**Affiliations:** 1 Institute for Quantitative Social Science, Harvard University, Cambridge MA 02138, USA; 2 Department of Humanities and Social Sciences in the Professions, Steinhardt School of Culture, Education and Human Development, New York University, USA; 3 Department of Global Health Policy, Graduate School of Medicine, University of Tokyo, Japan

## Abstract

**Background:**

Verbal autopsy analyses are widely used for estimating cause-specific mortality rates (CSMR) in the vast majority of the world without high-quality medical death registration. Verbal autopsies -- survey interviews with the caretakers of imminent decedents -- stand in for medical examinations or physical autopsies, which are infeasible or culturally prohibited.

**Methods and Findings:**

We introduce methods, simulations, and interpretations that can improve the design of automated, data-derived estimates of CSMRs, building on a new approach by King and Lu (2008). Our results generate advice for choosing symptom questions and sample sizes that is easier to satisfy than existing practices. For example, most prior effort has been devoted to searching for symptoms with high sensitivity and specificity, which has rarely if ever succeeded with multiple causes of death. In contrast, our approach makes this search irrelevant because it can produce unbiased estimates even with symptoms that have very low sensitivity and specificity. In addition, the new method is optimized for survey questions caretakers can easily answer rather than questions physicians would ask themselves. We also offer an automated method of weeding out biased symptom questions and advice on how to choose the number of causes of death, symptom questions to ask, and observations to collect, among others.

**Conclusions:**

With the advice offered here, researchers should be able to design verbal autopsy surveys and conduct analyses with greatly reduced statistical biases and research costs.

## Introduction

Estimates of cause-specific morality rates (CSMRs) are urgently needed for many research and public policy goals, but high quality death registration data exists in only 23 of 192 countries [1]. Indeed, more than two-thirds of deaths worldwide occur without any medical death certification [2]. In response, researchers are increasingly turning to verbal autopsy analyses, a technique "growing in importance" [3]. Verbal autopsy studies are now widely used in the developing world to estimate CSMRs, disease surveillance, and sample registration [4-6], as well as risk factors, infectious disease outbreaks, and the effects of public health interventions [7-9].

The idea of verbal autopsy analyses is to ask (usually around 10-100) questions about symptoms (including some signs and other indicators) of the caretakers of randomly selected decedents and to infer from the answers the cause of death. Three approaches have been used to draw these inferences. We focus on the fourth and newest approach, by King and Lu, which requires many fewer assumptions [10]. We begin by summarizing the main existing approaches. We then discuss our main contribution, which is in the radical new (and much easier) ways of writing symptom questions, weeding out biased symptoms empirically, and choosing valid sample sizes.

The first is *physician review*, where a panel of (usually three) physicians study the reported symptoms and assign each death to a cause, and then researchers total up the CSMR estimates [11]. This method tends to be expensive, time- consuming, unreliable (in the sense that physicians disagree over a disturbingly large percentage of the deaths), and incomparable across populations (due to differing views of local physicians); in addition, the reported performance of this approach is often exaggerated by including information from medical records and death certificates among the symptom questions, which is unavailable in real applications [12]. The second approach is *expert systems*, where a decision tree is constructed by hand to formalize the best physicians' judgments. The result is reliable but can be highly inaccurate with symptoms measured with error [13,14].

The third approach is *statistical classification *and requires an additional sample of deaths from a medical facility where each cause is known and symptoms are collected from relatives. Then a parametric statistical classification method (e.g., multinomial logit, neural networks, or support vector machines) is trained on the hospital data and used to predict the cause of each death in the community [15]. These methods are unbiased only if one of two conditions hold. The first is that the symptoms have 100% sensitivity and specificity. This rarely holds because of the inevitable measurement error in survey questions and the fact that symptoms result from rather than (as the predictive methods assume) generate the causes that lead to death. A second condition is that the symptoms represent all predictive information and also that everything about symptoms and the cause of death is the same in the hospital and community (technically, the symptoms span the space all predictors of the cause of death and the joint distribution of causes and symptoms are the same in both samples [16]). This condition is also highly unlikely to be satisfied in practice.

Like statistical classification, the King-Lu method [10] is data-derived and so requires less qualitative judgment; it also requires hospital and community samples, but makes the much less restrictive assumption that only the distribution of symptoms for a given cause of death is the same in the hospital and community. That is, when diseases present to relatives or caregivers in similar ways in the hospital and community, then the method will give accurate estimates of the CSMR. This is true even when the symptom questions chosen have very low (but nonzero) sensitivity and specificity, when these questions have random measurement error, when the prevalence of different symptoms and the CSMR differ dramatically between the hospital and community, or if the data from the hospital are selected via case-control methods. Case-control selection, where the same number of deaths are selected in the hospital for each cause, can save considerable resources in conducting the survey, especially in the presence of rare causes of death in the community. The analysis (with open source and free software) is easy and can be run on standard desktop computers. In addition, so long as the symptoms that occur with each cause of death do not change dramatically over time (even if the prevalence of different causes do change), the hospital sample can be built up over time, further reducing the costs.

Conceptually, the King-Lu method involves four main advances (see Appendix A for a technical summary). First, King-Lu estimates the CSMR, which is the main quantity of interest in the field, rather than the correct classification of any individual death. The method generates unbiased estimates of the CSMR even when the percent of individual deaths that can be accurately classified is very low. (The method also offers a better way to classify individual deaths, when that is of interest; see Appendix A and [[10], Section 8].) Second is a generalization to multiple causes of the standard "back calculation" epidemiological correction for estimating the CSMR with less than perfect sensitivity and specificity [17,18]. The third switches "causes" and "effects" and so it properly treats the symptoms as consequences rather than causes of the injuries and diseases that lead to death. Since symptoms are now the outcome variables, it easily accommodates random measurement error in survey responses. The final advance of this method is dropping all parametric modeling assumptions and switching to a fully nonparametric approach. The result is that we merely need to tabulate the prevalence of different symptom profiles in the community and the prevalence of different symptom profiles for each cause of death in the hospital. No modeling assumptions, not much statistical expertise, and very little tweaking and testing are required to use the method.

Building on [10], we now develop methods that minimize statistical bias in Section and inefficiency in Section 0.4 by appropriately choosing symptom questions, defining causes of death, deciding how many interviews to conduct in the hospital and community, and adjusting for known differences between samples. Software to estimate this model is available at http://gking.harvard.edu/va.

## Avoiding Bias

[10] indicates how to avoid bias from a statistical perspective. Here, we turn these results and our extensions of them into specific, practical suggestions for choosing survey questions. We do this first via specific advice about designing questions (Section 0.1), then in a section that demonstrates the near irrelevance of sensitivity and specificity, which most previous analyses have focused on (Section 0.2), and finally via a specific method that automates the process of weeding out questions that violate our key assumption (Section 0.3).

### 0.1 Question Choice

The key to avoiding bias is ensuring that patients who die in a hospital present their symptoms in the verbal autopsy survey instrument (as recorded by relatives and caregivers) in the same way as do those in the community (or in other words that the assumption in Equation 3 holds). As such, it may seem like the analyst is at the mercy of the real world: either diseases present the same way in the two locations or they do not. In fact, this is not the case, as the analyst has in the choice of symptom questions a powerful tool to reduce bias. In other words, how the patient presents symptoms to relatives and caregivers is only relevant inasmuch as the analyst asks about them. That means that avoiding bias only requires not posing symptom questions likely to appear different to those in the two samples (among those who cared for people dying of the same cause).

For an example of a violation of this assumption, consider that relatives of those who died in a medical facility would be much more likely than their counterparts in the community to know if the patient under their care suffered from high blood pressure or anemia, since relatives could more easily learn these facts only from medical personnel in a hospital than in the community without proper ways of measuring these quantities. Avoiding questions like these can greatly increase the accuracy of estimates. In contrast, the respondent noting whether they observed the patient bleeding from the nose would be unlikely to be affected by a visit to a hospital, even under instruction from medical personnel.

This fundamental point suggests avoiding any symptom questions about concepts more likely to be known by physicians and shared with patients or taught to caregivers than known by those in the community who were unlikely to have been in contact with medical personnel. We should therefore be wary of more sophisticated, complicated, or specialized questions likely to be answerable only by those who have learned them from medical personnel. The questions a relative is even likely to understand and answer accurately are not those which a physician might determine from a direct physical examination. *Trying to approximate the questions physicians ask themselves is thus likely to lead *to bias. In contrast, finding questions respondents are easily able to answer, and likely to give the same answers despite their experience in a medical facility, is best.

It is important to understand how substantially different these recommendations are from the way most have gone about selecting verbal autopsy survey questions. Most studies now choose questions intended to have the highest possible sensitivity and specificity. At best, this emphasis does not help much, because they are not required for accurate CSMR inferences, a point we demonstrate in the next section. However, this emphasis can also lead to huge biases when symptom questions with highest sensitivity and specificity are those that are closest to questions physicians would ask themselves, medical tests, or other facts which relatives of the deceased learn about about when in contact with medical personnel. (A related practical suggestion for reducing bias is to select a hospital as similar as possible to the community population. In most cases, physical proximity to large portions of the community will be helpful, but the main goal should be selecting a medical facility which has patients that present their symptoms in similar ways in hospital as in the community for each cause of death.)

### 0.2 The Near Irrelevance of Sensitivity and Specificity

Verbal autopsy researchers regularly emphasize selecting symptom questions based on their degree of sensitivity and specificity. It is hard to identify a study that has selected symptom questions in any other way, and many criticize verbal autopsy instruments for their low or variable levels of sensitivity and specificity across data sets and countries. This emphasis may be appropriate for approaches that use statistical classification methods, as they require 100% sensitivity and specificity, but such stringent and unachievable requirements are not needed with the King-Lu approach. We now provide evidence of this result.

We begin by generating data sets with 3,000 sampled deaths in the community and the same number in a nearby hospital (our conclusions do not depend on the sample size). The data consist of answers to 50 symptom questions -- which we use in subsets of 20, 30, and 50 -- and 10 causes of death. Because we generated the data, we know both the answers to the symptom questions and the cause of death for everyone in both samples; however, we set aside the causes of death for those in the community, which we ordinarily would not know, and use them only for evaluation after running each analysis.

We first generate a "high sensitivity" symptom, which we shall study the effect of. It has 100% sensitivity for the first cause of death and 0% for predicting any other cause of death (i.e., it also has high specificity). (The prevalence of this first cause of death is 20% overall.) Every other symptom has a maximum of 30% sensitivity for any cause of death. For each of 20, 30, and 50 symptoms, we generate 80 data sets with and without this high sensitivity symptom (each with 3,000 samples from the hospital and 3,000 from the community). In each data set, we estimate the CSMR. We then average over the 80 results for each combination of symptoms and compare the average of these estimates to the truth. This averaging is the standard way of setting aside the natural sampling variability and focusing on systematic patterns.

The results appear in Table 1, with different numbers of symptoms given in separate columns. The first two rows of the table give the difference in the proportion of deaths estimated to be in the first category with (for the first row) and without (for the second) the high sensitivity symptom. Clearly the difference between the numbers in the first two rows is trivial, indicating the near irrelevance of including this symptom. The second pair of rows in the table give the absolute error in estimating the effects for all causes of death. Again, the results show the irrelevance of including this high sensitivity symptom. (The numbers in the tables are proportions, so that 0.0128 represents a 1.28 percentage point difference between the estimate and the truth.)

**Table 1:** Absolute error rates with and without a symptom that has very high sensitivity for the first cause of death.

	Number of Symptoms		
	20	30	50
*Absolute Error for First Cause of Death*			
With high sensitivity symptom	0.0128	0.0120	0.0123
Without high sensitivity symptom	0.0128	0.0124	0.0124
*Mean Absolute Error*			
With high sensitivity symptom	0.0090	0.0081	0.0084
Without high sensitivity symptom	0.0092	0.0082	0.0085

The first and second row of each pair are very similar, which illustrates the irrelevance of finding symptoms with high sensitivity.

Searching for high sensitivity symptoms is thus at best a waste of time. The essential statistical reason for this is that symptoms do not cause the diseases and injuries that result in death. Instead, the symptoms are consequences of these diseases and injuries. As such, assessing whether the symptoms have high sensitivity and specificity for predicting something they do not predict (or cause) in the real world, and are not needed to predict with the method, is indeed irrelevant.

The King-Lu method works by appropriately treating symptoms as consequences of the disease or injury that caused death. Even if they were available, having symptoms that approximate highly predictive bioassays is neither necessary nor even very helpful. Any symptom which is a consequence of one or more of the causes of death can be used to improve CSMR estimates, even if it has random measurement error. So long as a symptom has some, possibly low-level, relationship to the causes of death, it can be productively used. Even apparently tertiary, behavioral, or non-medical symptoms that happen to be consequences of the illness can be used so long as they are likely to be reported in the same way regardless of whether the death occurred in a hospital or the community.

### 0.3 Detecting Biased Symptom Questions

Suppose we follow the advice offered on selecting symptom questions in Section 0.1, but we make a mistake and include a question which unbeknownst to us induces bias. An example would be a symptom which, for a given cause of death, is overreported in the hospital vs the community. Appendix B shows that in some circumstances it's possible to detect this biased symptom question, in which case we can remove it and rerun the analysis without the offending question. Since many verbal autopsy instruments include a large number of questions, this procedure could be used to eliminate questions without much cost. In this section, we present analyses in simulated and real data that demonstrate the effectiveness of this simple procedure.

We thus conduct a simulations where different numbers of symptom questions are "misreported" -- that is, reported with different frequencies in the hospital and community samples for given causes of death. We generate two sets of data, one with 3,000 deaths in each of the hospital and community samples and the other with 500 deaths in each; both have 10 different causes of death, with distribution *D *= (0.2, 0.2, 0.2, 0.1, 0.05, 0.05, 0.05, 0.05, 0.05, 0.05). The CSMF distribution of *D *in the hospital is uniform (0.1 for each cause) and in the community is *D *= (0.2, 0.2,..., 0.2, 0.05). Table 2 summarizes the pattern of misreporting we chose. In this table, the first row indicates which symptom number is to be set up as misreported when applicable. The second row gives the extent of the violation of the key assumption of the King-Lu method (in Equation 3) -- the percentage point difference between the symptom's marginal distribution in the community and hospital samples. For example, where three symptoms are misreported, we generate data using the first three columns of Table 2 and so the marginal prevalence of the first, fifth, and 10th symptoms in the community sample are set to be different from the hospital according to the first three elements of the second column. When five symptoms are misreported, the distribution of the first, fifth, 10th, 11th and 15th symptoms will be set to be different in the degree as indicated, etc.

**Table 2:** List of Misreported Symptoms

Symptom	1	5	10	11	15	20	21	25	30	31
Misreport	30%	-30%	-50%	30%	*-*30%	30%	-30%	-50%	30%	-30%

We first give our large sample and small sample results and then give a graphic illustration of the bias-efficiency trade-offs involved in symptom selection.

#### Larger Sample Size

For the large (*n *= 3, 000) simulation, Table 3 summarizes the results of symptom selection for each different simulation. The first two columns indicate, respectively, the total number of symptoms and the number of biased symptoms included in the community sample. The third column indicates the number of symptoms flagged (at the 5% significance level), using the selection procedure proposed in Appendix B. The final column indicates the number of biased symptoms that are correctly identified.

**Table 3:** Performance of the Symptom Selection Method with *n *= 3, 000

symptoms	biased	flagged	correct
10	3	0	0
20	3	3	3
30	3	3	3
50	3	3	3
20	5	5	5
30	5	5	5
50	5	6	5
50	10	10	10

Table 3 indicates that our symptom selection procedure is highly accurate. Except the first simulation, which has disproportionately many misreported symptoms (three out of 10), our proposed method correctly selects nearly all the biased symptoms. There is only one false positive case, but if we were to change the joint significance level to 0.01, this symptom would no longer being incorrectly selected.

#### Smaller Sample Size

Our smaller (*n *= 500) simulation necessarily results in larger CSMR variances. This, in turn, affects the power of the method described in Appendix B to detect biased symptoms. And at the same time it makes it more costly to discard unbiased symptoms. As shown in Table 4, we miss some symptoms at the 5% level. If we relax the type I error rate to 10%, more biased symptoms are detected. The table also conveys the additional variation in our methods due to the smaller sample size.

**Table 4:** Performance of the Symptom Selection Method with *n *= 500

		5% error rate		10% error rate	
symptoms	biased	flagged	correct	flagged	correct
10	3	0	0	1	1
20	3	3	3	3	3
30	3	2	2	5	3
50	3	3	3	5	3
20	5	2	2	3	3
30	5	3	3	4	4
50	5	4	4	4	4
50	10	6	3	6	3

#### Bias-Efficiency Trade-offs

Although these results are encouraging, in real-world data, a trade-off always exists between choosing a lower significance level to ensure that all biased symptoms are selected at a cost of increasing the false positive rate, vs. choosing a higher significance level to reduce the false positive rate at the cost of missing some biased symptoms. In this section, we illustrate how different sample sizes can affect the decision about the significance level in practice.

**Figure 1:**
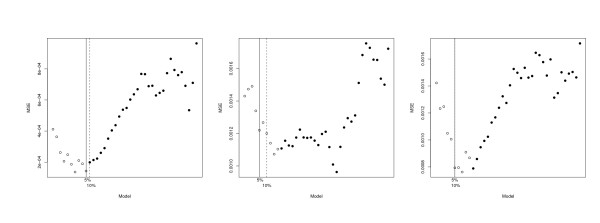
**For sample sizes of 3,000 (left graph), 1,000 (middle), and 500 (right), the figure gives the mean square error as symptoms are removed using our detection diagnostic**. The mean square error first declines, as bad symptoms are removed and bias drops, and then increases, as unbiased symptoms are dropped and variance increases. The procedure selects all biased symptoms (open circles) before unbiased symptoms (solid disks). The vertical line indicates where our automated procedure would indicate that we should stop.

We now choose "large" (3, 000), "medium" (1, 000), and "small" (500) samples for each of the community and hospital samples. In each, 10 of 50 symptoms are biased. We then successively remove symptoms all the way up to the 50% signficance level criterion (to see the full consequence of dropping too many symptoms). Figure 1 gives the results in a graph for each sample size separately, (large, medium, and small sample sizes from left to right). Each graph then displays the mean square error (MSE) between the true and estimated cause-of-death distribution for each run of the model vertically, removing more and more symptoms as from the left to the right along the horizontal axis of each graph. (In real applications, we would never be able to compute the MSE, because we do not observe the true cause of death, but we can do it here for validation because the data are simulated.) That is, all symptoms are included for the first point on the left, and each subsequent point represents a model estimated with an additional symptom dropped, as selected by our procedure. Circles are plotted for models where a biased symptom was selected and solid disks are plotted for unbiased symptoms.

Three key results can be seen in this figure. First, for all three sample sizes and corresponding graphs, all biased symptoms are selected before any unbiased symptoms are selected, without a single false positive (which can be seen because all circles appear to the left of all solid disks).

Second, all three figures are distinctly U-shaped. The reason can be seen beginning at the left of each graph with a sample that includes biased symptoms. As our selection procedure detects and deletes these biased symptoms one at a time, the MSE drops due to the reduction in bias of the estimate of the CSMR. Dropping additional symptoms after all biased ones are dropped, which can occur if the wrong significance level is chosen, will induce no bias in our results but can reduce efficiency. If few unbiased symptoms are deleted, little harm is done since most verbal autopsy data sets have many symptoms and so enough efficiency to cover the loss. However, if many more unbiased symptoms are dropped, the MSE starts to increase because of inefficiency, which explains the rest of the U-shape.

Finally, in addition to choosing the order in which symptoms should be dropped, our automated selection procedure also suggests a stopping rule based on the user-choice of a significance level. The 5% and 10% significance level stopping rules appear on the graphs as solid and dashed lines. These do not appear exactly at the bottom of the U-shape, cleanly distinguishing biased from unbiased symptoms, but for all three sample sizes the results are very good. In all cases, using this stopping rule would greatly improve the MSE of the ultimate estimator. Our procedure thus seems to do work reasonably well.

#### Empirical Evidence from Tanzania

**Figure 2:**
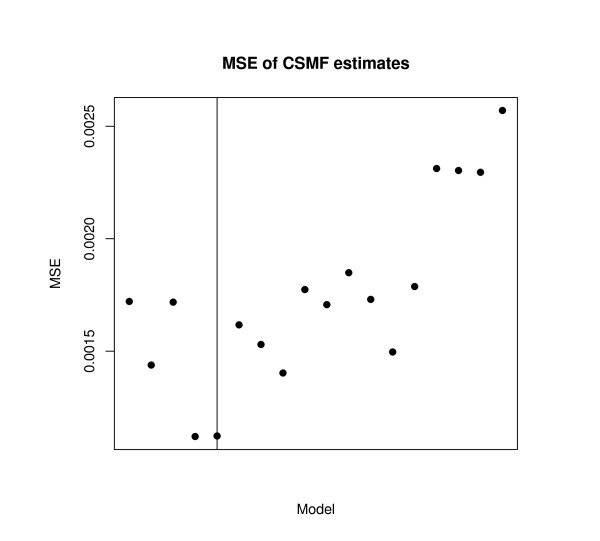
**Validation of Mean Square Error in Tanzania, where the true cause of death is known in both samples**.

Finally, we apply the proposed validation method to a real data example where we happen to observe the true causes of death in both samples [19]. These data from Tanzania have 12 causes of death and 51 symptoms questions but a small sample of only 282 deaths in the community sample and 1,261 in the hospital. Applying our symptom selection method, at the 5% level, four symptoms would be deleted, which in fact corresponds to the smallest MSE along the iterative model selection process. Figure 2 gives these results.

To illustrate this example in more details, Table 5 shows how the mean square error changes when specific symptoms were sequentially selected, And in Table 6 we present the change in each cause-specific mortality fraction estimate as these four symptoms are sequentially removed.

**Table 5:** List of symptoms that are sequentially removed from the analysis.

	prevalence		
symptoms	hospital	community	mean square error
-	-	-	0.0016
fever	72.6	45.4	0.0013
pale	33.1	17.4	0.0018
confused	30.8	14.5	0.0012
wheezing	8.3	21.3	0.0012
vomit	49.0	35.5	0.0015
difficult-swallow	18.9	7.4	0.0015
diarrhoea	29.8	20.9	0.0014
chest-pain	43.9	33.0	0.0017
pins-feet	14.6	8.5	0.0015
many-urine	8.4	5.3	0.0016
breathless-flat	28.2	35.5	0.0015
pain-swallow	15.5	6.7	0.0015
mouth-sores	22.7	13.8	0.0018
cough	50.0	38.7	0.0020
body-stiffness	6.9	2.5	0.0021
puffiness-face	11.7	11.3	0.0025
breathless-light	34.9	33.3	0.0027

**Table 6:** Cause-specific mortality fraction estimates when the first four symptoms are removed.

			sequentially removed			
cause of death	all 51 symptoms	fever	pale	confused	wheezing	true
HIV	0.146	0.177	0.164	0.185	0.190	0.227
Malaria	0.073	0.082	0.084	0.091	0.101	0.089
Tuberculosis	0.063	0.071	0.086	0.073	0.077	0.035
Infectious diseases	0.058	0.047	0.050	0.044	0.044	0.028
Circulatory diseases	0.225	0.215	0.159	0.163	0.160	0.163
Maternal diseases	0.035	0.026	0.030	0.033	0.032	0.035
Cancer	0.042	0.033	0.033	0.039	0.041	0.092
Respiratory diseases	0.070	0.079	0.070	0.073	0.053	0.046
Injuries	0.039	0.034	0.028	0.023	0.031	0.050
Diabetes	0.113	0.108	0.150	0.133	0.139	0.053
Other diseases I*	0.023	0.031	0.030	0.027	0.027	0.032
Other diseases II	0.170	0.164	0.173	0.159	0.162	0.149

*Note: "other disease I" includes diseases in residual category that are related to internal organs

The first three symptoms which were sequentially dropped -- fever, pale, and confused -- are so non-specific that they were almost unable to distinguish all of the 12 causes clinically, with the possible exception of deaths from injuries. Although high levels of specificity are not necessary for our method, we do require that it is above zero. Wheezing is a distinctive clinical symptom of an airway obstruction, typically observed in patients with asthma or bronchitis, and would be useful to identify deaths from such causes. However, our method suggests that it was highly biased. In fact, Table 5 indicates that there was a substantial difference in the prevalence of wheezing between community and hospital deaths (8.3% and 21.3%, respectively). This would mean that the wheezing observed in the community may be highly biased due to the respondents' difficulty in understanding the symptom correctly. Other symptoms which can be considered unbiased, such as vomiting, difficulty in swallowing, and chest pain, are all clinically useful in distinguishing the 12 causes of death, and the difference in prevalence between community and hospital deaths was much smaller.

### 0.4 Adjusting for Sample Differences

Suppose the key assumption of the King-Lu method is violated because of known differences in the hospital and community samples. For example, it may be that through outreach efforts of hospital personnel, or because of sampling choices of the investigators, children are overrepresented in the hospital sample. Even if the key assumption applies within each age group, diseases will present differently on average in the two samples because of the different age compositions. When it is not feasible to avoid this problem by desiging a proper sampling strategy, we can still adjust ex post to avoid bias, assuming the sample is large enough. The procedure is to estimate the distribution of symptoms for each cause of death within each age group separately, instead of once overall, and then to weight the results by the age distribution in the community.

As Appendix A shows in more detail, this procedure can also be applied to any other variables with known distributions in the community, such as sex or education. Since variables like these are routinely collected in verbal autopsy interviews, this adjustment should be easy and inexpensive, and can be powerful.

## Reducing Inefficiencies

We now suppose that the analyst has chosen symptom questions as best as possible to minimize bias, and study what can be done to improve statistical efficiency. Efficiency translates directly into our uncertainty estimates about the CSMR, such as the standard error or confidence interval, and improving efficiency translates directly into research costs saved. We study efficiency by simulating a large number of data sets from a fixed population and measure how much estimates vary. We then study how efficiency responds to changes in the number of symptom questions (10, 20, 30, and 50), size of the hospital sample (500, 1,000, 2,000, 3,000, and 5,000), size of the community sample (1,000, 2,000, 3,000, 5,000, and 10,000), and number of chosen causes of death (5, 10, and 15). Causes of death in the hospital are assumed to be constructed via a case-control methods, with uniform CSMR across causes. The CSMR in the community for each cause of death has some prevalent causes and some rarer causes.

**Figure 3:**
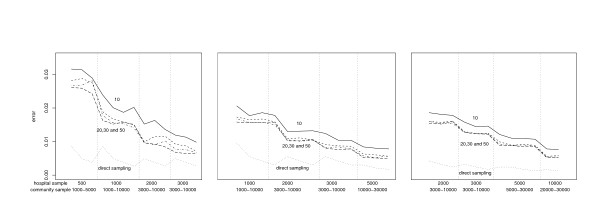
**Simulation Results for 5 (left graph), 10 (middle), and 15 (right) causes of death**.

For each combination of symptoms, hospital and community samples, and number of causes of death, we randomly draw 80 data sets. For each, we compute the absolute error between the King-Lu estimate and the truth per cause of death, and finally average over the 80 simulations. This *mean absolute error *appears on the vertical axis of the three graphs (for 5, 10, and 15 causes of death reading from left to right) in Figure 3. The horizontal axis codes both the hospital sample size and, within categories of hospital sample size, the community sample size. Each of the top four lines represent different numbers of symptoms.

The lower line, labeled "direct sampling," is based on an infeasible baseline estimator, where the cause of each randomly sampled community death is directly ascertained and the results are tabulated. The error for this direct sampling approach is solely due to sampling variability and so serves as a useful lower error bound to our method, which includes both sampling variability and error due to extrapolation between the hospital and population samples. No method with the same amount of available information could ever be expected to do better than this baseline.

Figure 3 illustrates five key results. First, the mean absolute error of the King-Lu method is never very large. Even in the top left corner of the figures, with 500 deaths in the hospital, 1,000 in the community, and only 10 symptom questions, the average absolute error in the cause-of-death categories is only about 2 percentage points, and of this 0.86, of a percentage point is due to pure irreducible sampling variability (see the direct sampling line).

Second, increasing the number of symptom questions, regardless of the hospital and community sample sizes, reduces the mean absolute error. So more questions are better. However, the advantage in asking more than about 20 questions is fairly minor. Asking five times more questions (going from 10 to 50) reduces the mean absolute error only by between 15% and 50%. Our simulation is using symptom questions that are statistically independent, and so more questions would be needed if different symptoms are closely related; however, additional benefit from more symptoms would remain small.

Third, the mean absolute error drops with the number of deaths observed in the community, and can be seen for each panel separated by vertical dotted lines in each graph. Within each panel, the slope of each line drops at first and then continues to drop but at a slower rate. This pattern reflects the diminishing returns of collecting more community observations, so increasing from 5,000 to 10,000 deaths does not help as much as increasing the sample size from 1,000 to 3,000.

Fourth, mean absolute error is also reduced by increasing the hospital sample size. Assuming since data collection costs will usually keep the community sample larger than the hospital sample, increasing the hospital sample size will always help reduce bias. Moreover, within these constraints, the error reduction for collecting an extra hospital death is greater than that in the community. The reason for this is that the method estimates only the marginal distribution of symptom profile prevalences from the community data, whereas it estimates this distribution within each category of deaths in the hospital data. It is also true that the marginal gain in the community sample is constrained to a degree by the sample size in the hospital; the reason is that combinations of symptoms in the community can only be analyzed if examples of them are found in the hospital.

And finally, looking across the three graphs in Figure 3, we see that the mean absolute error per cause drops slightly or stays the same as we increase the number of causes of death. Of course, this also means that with more causes, the total error is increasing as the number of causes increase. This is as it should be because estimating more causes is a more difficult inferential task.

The results in this section provide detailed information on how to design verbal autopsy studies to reduce error. Researchers can now pick an acceptable threshold level for the mean absolute error rate (which will depend on the needs of their study and what they want to know) and then choose a set of values for the design features that meets this level. Since the figure indicates that different combinations of design features can produce the same mean absolute error level, researchers have leeway to choose from among these based on convenience and cost-effectiveness. For example, although the advantage of asking many more symptom questions is modest, it may be that in some areas extra time spent with a respondent is less costly than locating and traveling to another family for more interviews, in which case it may be more cost-effective to ask 50 or more symptom questions and instead reduce the number of interviews. Figure 3 provides many other possibilities for optimizing scarce resources.

## Discussion

Despite some earlier attempts at promoting standard tools [20], which have now been adopted by various users including demographic surveillance sites under the INDEPTH Network [19,21,22], little consensus existed for some time on core verbal autopsy questions and methods. In order to derive a set of standards and to achieve a high degree of consistency and comparability across VA data sets, a recent WHO-led expert group recently systematically reviewed, debated, and condensed the accumulated experience and evidence from the most widely-used and validated procedures. This process resulted in somewhat more standardized tools, which are now adopted by various users, including demographic surveillance sites.

Verbal autopsy methodologies are still evolving: several key areas of active and important research remain. A research priority must be to carry out state-of-the-art validation studies of the new survey instruments in multiple countries with varying mortality burden profiles, using the methods discussed and proposed here. Such a validation process would contribute to the other areas of research, including further optimization of items included in questionnaires; replicable and valid automated methods for assigning causes of death from VA that remove human bias from the cause-of-death assignment process; and such important operational issues as sampling methods and sizes for implementing VA tools in research demographic surveillance sites, sample or sentinel registration, censuses, and household surveys.

With the advice we offer here for writing symptom questions, weeding out biased questions, and choosing appropriate hospital and community sample sizes, researchers should be able to greatly improve their analyses, reducing bias and research costs. We encourage other researchers and practitioners to use these tools and methods, to refine them, and to develop others. With time, this guidance and experience ought to better inform the VA users, and enhance the quality, comparability, and consistency of causespecific mortality rates throughout the developing world.

## Appendix A

### The King-Lu Method and Extensions

We describe here the method of estimating the CSMR offered in [10]. We give some notation, the quantities of interest, a simple decomposition of the data, the estimation strategy, and a procedure for making individual classifications when useful.

#### Notation

Hospital deaths may be selected randomly, but the method also works without modification if they are selected via case-control methods whereby, for each cause, a fixed number of deaths are chosen. Case-control selection can greatly increase the efficiency of data collection. Deaths in the community need to be chosen randomly or in some representative fashion. Define an index *i *(*i *= 1,..., *n*) for each death in a hospital and ℓ (ℓ =1,..., *L*) for each death in the community. Then define a set of mutually exclusive and exhaustive causes of death, 1,..., *J*, and denote *D*_ℓ _as the observed cause for a death in the hospital and *D*_*i *_as the unobserved cause for a death in the community. The verbal autopsy survey instrument typically includes a set of *K *symptom questions with dichotomous (0/1) responses, which we summarize for each decedent in the hospital as a *K *× 1 vector ***S***_*i *_= {*S*_*i*1_,..., *S*_*iK*_} and in the community as ***S***_ℓ _= {*S*_ℓ1_,..., *S*_ℓ*K*_}.

#### Quantity of Interest

For most public health purposes, primary interest lies not in the cause of any individual death in the community but rather the aggregate proportion of community deaths that fall into each category: *P*(*D*) = {*P*(*D *= 1),..., *P*(*D *= *J*)}, where *P*(*D*) is a *J *× 1 vector and each element of which is a proportion: , where 1(*a*) = 1 if *a *is true and 0 otherwise. This is an important distinction because King-Lu gives approximately unbiased estimates of *P*(*D*) even if the percent of individual deaths correctly classified is very low. (We also describe below how to use this method to produce individual classifications, which may of course be of interest to clinicians.)

#### Decomposition

For any death, the symptom questions contain 2^*K *^possible responses, each of which we call a symptom profile. We stack up each of these profiles as the 2^*K *^× 1 vector ***S ***and write *P*(***S***) as the probability of each profile occurring (e.g., with *K *= 3 questions *P*(***S***) would contain the probabilities of each of these (2^3 ^= 8) patterns occurring in the survey responses: 000, 001, 010, 011, 100, 101, 110, and 111). *P*(***S***|*D*) as the probability of each of the symptom profiles occurring within for a given cause of death *D *(columns of *P*(***S***|*D*) corresponding to values of *D*). Then, by the law of total probability, we write(1)

and, to simplify, we rewrite Equation 1 as an equivalent matrix expression:(2)

where *P*(*D*) is a *J *× 1 vector of the proportion of community deaths in each category, our quantity of interest. Equations 1 and 2 hold exactly, without approximations.

#### Estimation

To estimate **P**(*D*) in Equation 2, we only need to estimate the other two factors and then solve algebraically. We can estimate *P*(***S***) without modeling assumptions by direct tabulation of deaths in the community (using the proportion of deaths observed to have each symptom profile). The key issue then is estimating *P*(***S***|*D*), which is unobserved in the community. We do this by assuming it is the same as in the hospital sample, *P*^*h*^(***S***|*D*):(3)

This assumption is considerably less demanding than other data-derived methods, which require the full joint distribution of symptoms and death proportions to be the same: *P*^*h*^(***S***, *D*) = *P*(***S***, *D*). In particular, if either the symptom profiles (how illnesses that lead to death present to caregivers) or the prevalence of the causes of death differ between the hospital and community -- *P*^*h*^(***S***) ≠ *P*(***S***) or *P*^*h*^(*D*) ≠ *P*(*D*)-- then other dataderived methods will fail, but this method can still yield unbiased estimates. Of course, if Equation 3 is wrong, estimates from the King-Lu method can be biased, and so finding ways of validating it can be crucial, which is the motivation for the methods offered in the text. (Several technical estimation issues are also resolved in [10]: Because 2^*K *^can be very large, they use the fact that (2) also holds for subsets of symptoms to draw multiple random subsets, solve (2) in each, and average. They also solve (2) for *P*(*D*) by constrained least squares to ensure that the death proportions are constrained to the simplex.)

#### Adjusting for Known Differences Between Hospital and Community

Let *a *be an exogneous variable measured in both samples, such as age or sex. To adjust for differences in *a *between the two samples, we replace our usual estimate of *P*^*h*^(***S***|*D*) with a weighted average of the same estimator applied within unique values of a, *P*^*h*^(***S***_*a*_|*D*_*a*_), times the community distribution, , and where the summation is over all possible values of *a*.

#### Individual Classification

Although the quantity of primary interest in verbal autopsy studies is the CSMR, researchers are often interested in classifications of some individual deaths. As shown in [[10], Section 8], this can be done with an application of Bayes theorem, if one makes an additional assumption not necessary for estimating the CSMR. Thus, if the goal is *P*(*D*_ℓ _= *j*|***S***_ℓ _= *s*), the probability that individual ℓ died of cause *j *given that he or she suffered from symptom profile *s*, we can calculate it by filling in the three quantities on the right side of this expression:(4)

First is *P*(*D*_ℓ _= *j*), the optimal estimate of the CSMR, given by the basic King-Lu procedure. The quantity *P*(***S***_ℓ _= *s*|*D*_ℓ _= *j*) can be estimated from the training set, usually with the addition of a conditional independence assumption, and *P*(***S***_ℓ _= ***s***_ℓ_) may be computed without assumptions from the test set by direct tabulation. (Bayes theorem has also been used in this field for other creative purposes [23].)

#### A Reaggregation Estimator

A recent article [12] attempts to build on King-Lu by estimating the CSMR with a particular interpretation of Equation 4 to produce individual classifications which they then reaggregate back into a "corrected" CSMR estimate. Unfortunately, the proposed correction is in general biased, since it requires two unnecessary and substantively untenable statistical assumptions. First, it uses the conditional independence assumption for estimating *P*(***S***_ℓ _= *s*|*D*_ℓ _= *j*) --useful for individual classification but unnecessary for estimating the CSMR. And second, it estimates *P*(***S***_ℓ _= ***s***) from the training set and so must assume that it is the same as that in the test set, an assumption which is verifiably false and unnecessary since it can be computed directly from the test set without error [[10], Section 8]. To avoid the bias in this reaggregation procedure to estimate the CSMR, one can use the original King-Lu estimator described above. Reaggregation of appropriate individual classifications will reproduce the same aggregate estimates.

## Appendix B

### A Test for Detecting Biased Symptoms

If symptom *k*, *S*_*k*_, is overreported in the community relative to the hospital for a given cause of death, then we should expect the predicted prevalence  -- which can be produced by but is not needed in the King-Lu procedure--to be lower than the observed prevalence *P*(*S*_*k*_). Thus, we can view *P*(*S*_*k*_) as data point in regression analysis and the misreported prevalence of the *k*th symptom, *P*(*S*_*k*_) as an outlier. This means that we can detect symptoms that might bias the analysis by examining model fit. We describe this procedure here and then give evidence and examples.

#### Test Procedure

Let  be the estimated community CSMRs via the King-Lu procedure, and then fit the marginal prevalence of the *k*th symptom in the community  (calculated as ; see Appendix A). Then define the prediction error as the residual in a regression as .

Under King-Lu,  is unbiased, and each *e*_*k *_is mean 0 with variance . Moreover,

is approximately *t *distributed with *K *- 1 degree freedom. If, on the other hand, *P*^*h*^(*S*|*D*) ≠ *P*(*S*|*D*), we would expect *t*_*k *_will have an expected value that deviates from zero.

Based on above observation, we propose a simple iterative symptom selection procedure. This procedure avoids the classic the "multiple testing problem" by applying a Bonferroni adjustment to symptom selection at each iteration. At the chosen significance level α, we can then assess whether a calculated value of *t*_*k *_indicates that the *k*^th ^symptom violates our key assumption and so is biased. Thus:

1. Begin with the set of all symptoms *S *= (*S*_1_,..., *S*_*K*_), and those to be deleted, *B*. Initialize *B *as the null set, and the number of symptoms in the estimation, *K*_0_, as *K*_0 _= *K*.

2. Estimate *P*(*D*) using the King-Lu method.

3. For symptom *k *(*k *= 1,..., *K*_0_), estimate , and calculate *t*_*k*_.

4. Find the critical value associated with level α under the *t *distribution with *K*_0 _- 1 degree freedom, . To ensure that the overall significance of the all symptoms that belong to *B *is less than α, use the Bonferroni adjustment for the significance level (the set of *t*_*k *_test statistics associated with the symptoms in set *B *are stochastically independent of each other since the models are run sequentially). The number of the multiple independent tests is counted as the number of elements already in the set *B *plus the one that is being tested. (Since the maximum |*t*_*k*_| at each step of symptom selection tends to decrease as more "bad" symptoms are removed, it is sufficient to check whether the maximum |*t*_*k*_| of the current model is greater or less than the critical value, .)

5. If the largest value of |*t*_*k*_|, namely |*t*_*k'*_|, is greater than the critical value , remove the corresponding *k' *th symptom. Then set *K*_0 _= *K*_0 _- 1 and add symptom *k' *to set *B*.

6. Repeat step 2-5 until no new symptoms are moved from *S *to *B*.

## Competing interests

The authors declare that they have no competing interests.

## Authors' contributions

GK, YL, and KS participated in the conception and design of the study and analysis of the results. YL wrote the computer code and developed the statistical test and simulations; GK drafted the paper. All authors read, contributed to, and approved the final manuscript.
